# Gastric Perforation with Omental Patch Repair: A Rare Complication of Pulmonary Resuscitation in COVID-19 Pneumonia

**DOI:** 10.1155/2020/8850739

**Published:** 2020-11-07

**Authors:** Vincent Marcucci, Ratul Bhattacharyya, Stephanie Yee, Jamshed Zuberi, Mark Ingram

**Affiliations:** ^1^School of Medicine, St. George's University, Grenada West Indies; ^2^Department of Surgery, St. Joseph's University Medical Center, 703 Main St. Paterson, NJ 07503, USA

## Abstract

A 71-year-old male, diagnosed with coronavirus disease 2019 (COVID-19), was admitted to the medical-surgical floor for supportive treatment. The patient received bag-mask ventilation (BMV) secondary to severe hypoxia and reendotracheal intubation in the hospital on day eleven. A chest X-ray following reintubation noted concern for intra-abdominal air. Significant abdominal distention and subsequent diagnostic imaging showed pneumoperitoneum and a possible perforation of the stomach. The patient underwent an exploratory laparotomy with omental patching for a gastric perforation. Amidst the height of the COVID-19 pandemic, several important findings have been made through the disease sequelae of this individual patient.

## 1. Introduction

Gastric perforation is a potentially life-threatening condition involving a full-thickness injury to the wall of the stomach, creating an intra-abdominal communication between the gastric lumen and the peritoneal cavity. Without prompt diagnosis and treatment, significant morbidity can result, including peritonitis, septic shock, and death [1]. The clinical presentation of this pathology classically involves severe abdominal pain with distention and accompanying nausea and vomiting [2]. These clinical sequelae are not as pronounced in the ICU population, thus complicating our efforts in diagnosis. The incidence of gastric perforation is commonly a result from penetrating trauma, interventional procedures, or an intrinsic gastric pathology (i.e., ulceration or malignancy) [3]. Barotrauma to the lungs and gastric insufflation causing vomiting and aspiration have been previously reported in patients under BMV [3]. However, the occurrence of gastric perforation with BMV has been rarely reported [4]. COVID-19 is an infectious disease caused by severe acute respiratory syndrome coronavirus 2 (SARS-CoV-2) [5, 6]. With the emergence of the novel COVID-19, the association with gastric pathology is unclear. We present a rare case of a 71-year-old patient who suffered from a gastric perforation during admission for severe respiratory distress, as well as other complications secondary to COVID-19-associated pneumonia.

## 2. Case Presentation

**Figure 1:**
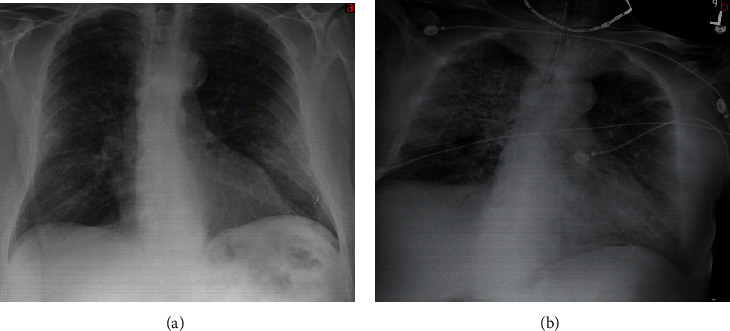
(a) CXR shows bilateral peripheral infiltrates. (b) Repeat CXR after 48 hrs demonstrating progressive lung infiltration.

A 71-year-old Caucasian male presented to our emergency department complaining of shortness of breath, dry cough, generalized malaise, and myalgias/arthralgias for one-week duration. His history was significant for hypertension, a previous inguinal hernia repair, and hemorrhoidectomy. Upon initial presentation, he was febrile (Tmax of 38.6C) and had a leukocyte count within normal limits. Chest X-ray (CXR) imaging revealed bilateral patchy peripheral infiltrates indicative of pneumonia (Figure 1(a)). A nasal swab upon presentation to the ER returned positive for COVID-19. Within 48 hours of admission, the patient's respiratory function continued to decline, and repeat CXR after ET placement showed progressive lung parenchymal consolidation (Figure 1(b)).

**Figure 2:**
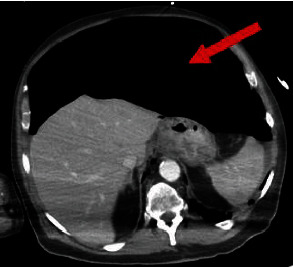
Free air within the peritoneal space indicated by the red arrow.

On the eleventh day of admission, the patient desaturated <65% due to ET cuff rupture requiring replacement. This prompted BMV, afterward, substantial abdominal distention was noted. Initial imaging with a CXR showed intraperitoneal free air, with a follow-up abdominal CT scan confirming massive free air in the abdomen (Figure 2). Surgical staffs were consulted, and the decision was made to bring the patient to the OR. Exploratory laparotomy was performed, identifying a 4 × 4cm anterior gastric perforation along lesser curvature.

## 3. Differential Diagnosis

Pneumoperitoneum (PP) from an intrathoracic route is the most commonly reported cause in nonsurgical intra-abdominal air collection, with an estimated incidence of approximately 7% in ICU patients [4]. In mechanically ventilated patients, PP has been strongly associated with pneumothorax and pneumomediastinum, likely from ruptured alveoli adjacent to the mediastinum or diaphragmatic dissection [4, 5]. However, PP should be considered in mechanically ventilated patients with underlying lung disease and frequent ventilator setting changes. Consideration should also be made for the development of PP from postthoracic trauma or iatrogenic lesions secondary to tracheal or esophageal procedures. Stress-related mucosal damage may be part of the diagnostic work up, especially when evaluating the possibility of PP in the intensive care unit. However, stress ulceration has been reported to occur within the first 24 hours of hospital admission in 75-100% of ICU patients [6], making this diagnosis less likely.

The diagnostic algorithm of determining the source of PP is likely related to the chronological relationship between the likeliest procedure of cause and its incidence [5]. In our case, the PP onset shortly after reendotracheal intubation and BMV is very suggestive. However, the incidence of intra-abdominal air collection as a manifestation of barotrauma is rare, which represents one of the novelties of the case at hand.

## 4. Treatment

COVID-19 currently has no established treatment guidelines. There is also no vaccine or proven preventative measures available at time. Mechanical ventilation may be necessary, as in our patient, who developed respiratory insufficiency refractory to oxygen therapy. In the event of associated gastric perforation, the treatment is emergent surgical intervention with open or laparoscopic repair [7].

## 5. Outcome and Follow-Up

Following the open exploratory laparotomy, our patient had no significant postoperative complications related to the procedure. However, the patient's respiratory function continued to decline. Upon review of surgical pathology, the patient's ulceration was found to be negative for a malignancy or H. Pylori infection. The specimen was found to have extensive hemorrhagic and ischemic mucosal necrosis. The patient's medication regimen did not include antacid therapies, and serum gastrin levels within normal limits were subsequently found. Biopsy from the perforation site at the gastric antrum showed significant focal congestion and mucosal necrosis, with abundant fibrin deposition in the submucosa. Possible etiologies include microthrombus due to hypercoagulability, tissue necrosis secondary to ischemia from critical illness, or possible stress ulcer at this time. Patient has no other indicated risks of stress ulcers, aside from critical illness requiring intubation. No past history of stress ulcers, reflux, or dyspepsia.

Two weeks after the surgery, our patient suffered acute hypoxia, refractory to maximum positive pressure ventilation. The patient's respiratory function continued to decline with ensuing cardiac arrest. This led to subsequent mortality, and he was pronounced as deceased in the ICU.

## 6. Discussion

We present a rare case of gastric perforation after receiving bag-mask ventilation and reintubation in a COVID-19-positive patient. Acute presentation of this disorder is usually found in adult patients with underlying gastrointestinal disorders such as chronic ulceration or malignancy [6–8]. We present an individual with no significant history of gastrointestinal disease. In this case, X-ray and CT imaging were able to provide a preliminary diagnosis. Surgical intervention with exploratory laparotomy allowed for definitive diagnosis and treatment.

Cases of gastric perforation are typically easily diagnosed via upright CXR or CT imaging and encompass one of the four mechanisms: ischemia (bowel obstruction, necrosis), infection (appendicitis, diverticulitis), erosion (malignancy, ulcerative disease), or physical disruption (trauma, iatrogenic injury) [9–11].

Several imaging modalities and laboratory tests are available to identify the incidence and etiology of a perforated viscus [11]. Abdominal and upright CXR are a fast and inexpensive method used to identify intra-abdominal free air. The most sensitive and specific test to diagnose a perforation is with abdominal/pelvic CT [11].

Pathology from our case revealed extensive hemorrhage and ischemic necrosis of the gastric antral mucosa. In COVID-19 patients with severe-to-critical respiratory insufficiency, pulmonary arterial oxygenation may be extensively impaired [12]. Severe and/or prolonged hypoxemia can result in diminished tissue perfusion and end organ failure. Hypoxemia, in conjunction with barotrauma from mechanical ventilation to the gastric mucosa, may result in acute transmural perforation, as seen in our patient [12, 13]. Gastric distention can induce or exacerbate ischemic changes to the mucosa by reducing blood flow to suboptimal levels. This change in oxygenation often predisposes to mucosal injury and possible perforation [13].

Atypical symptomatic presentation in COVID-19 patients has been previously reported. Gastrointestinal symptoms including abdominal pain, nausea, and vomiting have been found in up to 10% of cases [13, 14]. The presence of GI complaints and possible fecal-oral transmission of COVID-19 may be due to the use of ACE2 receptors present within the GI tract [15]. This utilization of ACE2 receptors in the gut by the novel SARS-CoV-2 has been hypothesized to decrease the expression of antimicrobial peptides, while altering gut microbial composition [15, 16].

With no presence of malignant disease or ulcerative process identified in our patient, etiologies involving erosive gastropathy are unlikely. Though dyspnea has been rarely reported in peptic ulceration, it is improbable for our patient with confirmed COVID-19 respiratory failure [17]. An infectious disease process can also be considered unlikely due to lack of relevant past medical history. Physical disruption due to overventilating with BMV and ischemic changes secondary to COVID-19 is the leading differential for our patient. However, it is interesting to note the possibility of gastric stress ulceration as a differential when the mucosal barrier is compromised in a hypoxic state much like our patient [6].

We suggest barotrauma from BMV as an appropriate differential for an intubated patient who developed acute abdominal distention after sudden respiratory compromise. This type of case should prompt immediate diagnostic imaging and surgical intervention to reduce morbidity and mortality.

## 7. Learning Points

The following are the learning points:

1. Gastric perforation as an appropriate leading differential for adult patients presenting with sudden onset of abdominal distention secondary to barotrauma
2. Understand the gastrointestinal correlation to COVID-19 and the associated morbidities in the adult population
3. States of hypercoagulation associated with COVID-19, are associated with such acute sequelae as ischemia of gastrointestinal organs and can cause subsequent perforation of gastrointestinal viscus
